# Role of TRPA1 in Tissue Damage and Kidney Disease

**DOI:** 10.3390/ijms22073415

**Published:** 2021-03-26

**Authors:** Chung-Kuan Wu, Ji-Fan Lin, Tzong-Shyuan Lee, Yu Ru Kou, Der-Cherng Tarng

**Affiliations:** 1 Division of Nephrology, Department of Internal Medicine, Shin-Kong Wu Ho-Su Memorial Hospital, Taipei 111, Taiwan; chungkuan.wu@gmail.com; 2 School of Medicine, College of Medicine, Fu-Jen Catholic University, New Taipei 242, Taiwan; 3 Precision Medicine Center, Department of Research, Shin-Kong Wu Ho-Su Memorial Hospital, Taipei 111, Taiwan; jifanlin@hotmail.com; 4 Department of Physiology, College of Medicine, National Taiwan University, Taipei 100, Taiwan; ntutslee@ntu.edu.tw; 5 Department of Institue of Physiology, School of Medicine, National Yang-Ming University, Taipei 112, Taiwan; yrkou@ym.edu.tw; 6 Department of Biological Science and Technology, National Chiao Tung University, Hsinchu 300, Taiwan; 7 Center for Intelligent Drug Systems and Smart Bio-devices (IDS2B), Hsinchu 300, Taiwan; 8 Institute of Clinical Medicine, National Yang-Ming University, Taipei 112, Taiwan; 9 Division of Nephrology, Department of Medicine, Taipei Veterans General Hospital, Taipei 112, Taiwan

**Keywords:** TRPA1, tissue damage, inflammation, kidney disease

## Abstract

TRPA1, a nonselective cation channel, is expressed in sensory afferent that innervates peripheral targets. Neuronal TRPA1 can promote tissue repair, remove harmful stimuli and induce protective responses via the release of neuropeptides after the activation of the channel by chemical, exogenous, or endogenous irritants in the injured tissue. However, chronic inflammation after repeated noxious stimuli may result in the development of several diseases. In addition to sensory neurons, TRPA1, activated by inflammatory agents from some non-neuronal cells in the injured area or disease, might promote or protect disease progression. Therefore, TRPA1 works as a molecular sentinel of tissue damage or as an inflammation gatekeeper. Most kidney damage cases are associated with inflammation. In this review, we summarised the role of TRPA1 in neurogenic or non-neurogenic inflammation and in kidney disease, especially the non-neuronal TRPA1. In in vivo animal studies, TRPA1 prevented sepsis-induced or Ang-II-induced and ischemia-reperfusion renal injury by maintaining mitochondrial haemostasis or via the downregulation of macrophage-mediated inflammation, respectively. Renal tubular epithelial TRPA1 acts as an oxidative stress sensor to mediate hypoxia–reoxygenation injury in vitro and ischaemia–reperfusion-induced kidney injury in vivo through MAPKs/NF-kB signalling. Acute kidney injury (AKI) patients with high renal tubular TRPA1 expression had low complete renal function recovery. In renal disease, TPRA1 plays different roles in different cell types accordingly. These findings depict the important role of TRPA1 and warrant further investigation.

## 1. Introduction

Renal failure is a major health problem worldwide [1]. Kidney diseases include acute kidney injury (AKI) and chronic kidney disease (CKD). Various ischemic and toxic substances can lead to kidney cell damage and inflammation-induced cell death, which subsequently results in AKI. AKI is a common and devastating pathologic condition and is defined as a rapid decrease in glomerular filtration rate [2]. AKI can be a reversible condition and has with high incidence and mortality; AKI is also the main cause of CKD [3] or end-stage renal disease (ESRD). Clinically, AKI is considered a significant risk factor for CKD and ESRD. For example, about half of recovered and discharged patients with hospital-associated AKI were diagnosed with CKD during the median follow-up period of 3.3 years [4]. The relative hazard risk of chronic dialysis among patients who recovered from dialysis-requiring AKI was 32.3 compared with controls [5].

Uremic toxins generated from high levels of metabolic end-products have become clinically relevant in CKD progression. These toxins are tightly related to many CKD-associated complications, such as hypertension, cardiovascular diseases, metabolic acidosis, anaemia, altered immune response, mineral and bone disturbances and neurological complications [6]. Cardiovascular dysfunctions and altered immune responses, which resulted in increased infections, have accounted for the risk of morbidity and mortality in CKD [7]. Inflammation and oxidative stresses play important roles in these conditions of CKD [8]. Moreover, patients with CKD typically suffer from chronic inflammation [9], and the dysfunction of the antioxidative systems worsens with the degree of renal function [10]. The treatment of inflammation and oxidative stresses is very important in CKD-associated complications. Inflammation is a crucial defence mechanism upon infection, and the dysregulation of inflammation may initiate a number of deleterious effects, including cytokine overproduction and an increase in proinflammatory and oxidative stress mediators [11]. Of interest, transient receptor potential ankyrin 1 (TRPA1), a member of the transient receptor potential channel (TRP) family, is a gatekeeper for inflammation and a molecular sentinel of tissue injury. In this work we will provide a comprehensive review of our current knowledge on this ion channel relative to kidney diseases.

## 2. TRPA1 as Mediator in Inflammatory Response

The immune system promotes protective responses and behaviour in acute inflammation in response to tissue injury. At the site of an injury, inflammatory agents are released to activate the neuronal and nonneuronal cells. The surrounding cells in the area of inflammation, such as keratinocytes, epithelial cells, and fibroblasts, release inflammatory mediators, including ATP, adenosine, bradykinin, leukotrienes, tumour necrosis factor α, interleukins, prostaglandins, proteases, and glutamate [12]. A subset of the primary sensory neurons is then activated to release inflammatory neuropeptides to promote extravasation of the plasma proteins, vasodilation, neutrophil accumulation, and hypersensitivity to thermal, chemical, and mechanical stimuli. Therefore, the sensory neurons are very important in sensing inflammation sites and promoting protective behaviour. Mounting lines of evidence have shown that TRPA1 plays a key role in regulating neuropeptide release and neurogenic inflammation [13].

**Figure 1:**
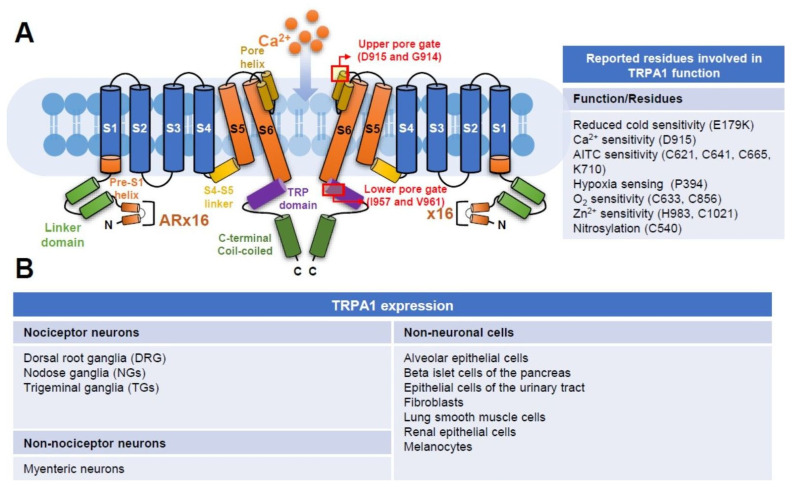
Structure and expression of TRPA1. (**A**) Key features of the ion channel TRPA1 mediating the cellular influx of calcium ions (Ca^2+^) are illustrated. Similar to other TRP channels, TRPA1 possesses a tetrameric structure, and a single pore is present along the central axis. Two subunits are shown here. Each subunit consists of six transmembrane alpha helices (S1–S6 domains), followed by two pore helixes, a TRP-like domain and an intracellular C-terminal domain. In the intracellular N-terminal, 16 ankyrin repeats (AR) are unique and speculated to contain the cysteine residues targeted by electrophilic TRPA1 activators. The molecular interactions of ligands with ARs may lead to conformational changes through the S4–S5 linker structure and subsequently open the channel. The location of reported upper and lower gates formed by residues D915/G914 and I957/V961, respectively, is shown. Protein residues reportedly involved in TRPA1 function or ion sensing are listed. (**B**) Expression of TRPA1 in selected neurons and non-neuronal cells.

The superfamily of TRP is composed of unique proteins expressed in almost every cell type; these channels include TRPC (Canonical), TRPV (Vanilloid), TRPM (Melastatin), TRPP (Polycystin), TRPML (Mucolipin), TRPN (NOMP-C) and TRPA (Ankyrin) according to their amino acid sequence homology [14]. TRPA1 is the only member of the TRPA subgroup in mammals. “A” in TRPA represents “ankyrin” repeats, which is composed of a 33-amino-acid motif in the N-terminal domain of this channel protein. In humans, 16 ankyrin repeats are found in TRPA1. These repeats are located within many proteins and function in protein–protein interactions; however, whether or not these repeats in TRPA1 mediate the interaction with other proteins remains unclear. The human TRPA1 gene, which is located in chromosome 8, comprises 73,635 bases and 29 exons (Gene ID: 8989) and encodes 1119 amino acids. Human TRPA1 contains a conserved six transmembrane α-helix (TM1-6) as other members of the TRP family. A re-entrant pore loop between TM5 and TM6 forms the central cavity of the channel and serves as two gates (Figure 1A). Ca^2+^ permeation is controlled by the upper gate (D915 and G914), and the permeation of the rehydrated cation is mediated by the lower gate (I957 and V961) consisting of two hydrophobic seals. A two-step mechanism of TRPA1 gates upon electrophile action and structurally conserved calcium control site was identified using cryo-EM technology [15]. In short, highly reactive cysteine (C621) and a nearby cysteine (C665) stabilize the loop in an activating configuration upon electrophile stimulation. When the loop is in an active status, the upper gate of TRPA1 widens the selectivity filter to enhance calcium permeability. The lower canonical gate at the cytoplasmic end of the channel then opens to allow the passing of cations, such as calcium. A conserved calcium coordination site comprising residues E788, Q791, Y799, N805, and E808 is found in TRPA1 as other calcium channels. Furthermore, several residues responsible for the function and ion sensing of TRPA1 have been reported. These findings provide a basic structural framework to understand how TRPA1 is controlled by endogenous and exogenous agents and pave the direction for future development of agonists or antagonists against TRPA1 (Figure 1B).

TRPA1 is a very attractive therapeutic target because it is robustly activated by a wide range of exogenous irritants that can cause pain and inflammation. For example, allyl isothiocyanate, cinnamaldehyde and allicin, which are found in mustard, cinnamon and garlic extracts, respectively, stimulate TRPA1. Air pollutants produced during the manufacturing of polymers, fertilizers, pesticides and other products can activate TRPA1 [16,17,18,19]. Common anaesthetics, such as isoflurane or lidocaine, also activate TRPA1 [20,21]. TRPA1 is targeted by endogenous inflammatory agents, such as reactive oxygen species (ROS). In response to tissue damage, cells release ROS, which subsequently causes lipid oxidation. The formation of reactive carbonyl species, including 4-hydroxynonenal and 4-oxononenal, stimulate TRPA1 directly [22,23]. TRPA1 is also activated by a fatty acid derivative, namely 15d-PGJ2, which is generated around inflammation sites to mediate inflammatory responses and sensitization [24]. Furthermore, the activation of TRPA1 can be modulated by G protein-coupled receptors (GPCRs) via second-messenger signalling cascades. In fact, TRPs are downstream effectors of GPCR nociceptive and pruritogenic signalling. This coordination forms the GPCR–TRP axis to sense itch, pain, neurogenic inflammation and analgesia [25] and allows TRPA1 to increase its repertoire of exogenous and endogenous stimuli. Extensive studies have found that TRPA1 is activated by numerous electrophilic and nonelectrophilic modulators, natural compounds, intracellular Ca^2+^, metals (such as Zn^2+^, Cd^2+^, and Cu^2+^), increased pH, cold and heat, mechanical stimulation, light, polyphosphates, phosphorylation, and interaction with TRPV1, ubiquitin hydrolase CYCL, PKA anchor protein AKAP5 and neuroendocrine secretory protein secretogranin-3 [26]. The demand for TRPA1 antagonists has increased because of the important role of TRPA1 in pain, inflammation, itch and respiratory disease. Natural compounds, such as camphor [27], borneol [28] and lutein [29] can inhibit TRPA1. Resolvins are endogenously produced from omega-3 polyunsaturated fatty acid and are anti-inflammatory and proresolving lipid molecules. At the submicromolar level, resolvin D1 inhibits cinnamaldehyde-activated TRPA1 [30]. The first synthetic antagonist of TRPA1 was developed in 2007 and named HC-030031 [31]. Various potent TRPA1 inhibitors are now available from different pharmaceutical companies, including Hydra Biosciences, Abbot, AMGEN, Pfizer, Glenmark and Renovis. On the basis of genetic mutation, knockout animal studies and preclinical studies using small molecule antagonists, TRPA1 remains an attractive target for pain, dermatological diseases and respiratory diseases. Therefore, further development of TRPA1 antagonists for clinical use is still warranted [32].

## 3. The Role of TRPA1 on Neuron and Non-Neuron Tissue Damages

Tissue damage, caused by toxin, disease, or trauma, evokes an inflammatory response at the site of injury to lessen harmful stimuli, mediate tissue repair and protect tissues from further damage. However, alterations in the haemostatic balance between the nociceptive and immune system reinforce the response to damaged signals, leading to chronic inflammation-related diseases, such as asthma, itch, pain, rheumatoid arthritis, and colitis. The TRPA1 channel is well known as a sensor of cellular stress, inflammation and tissue damage [13,33].

**Figure 2:**
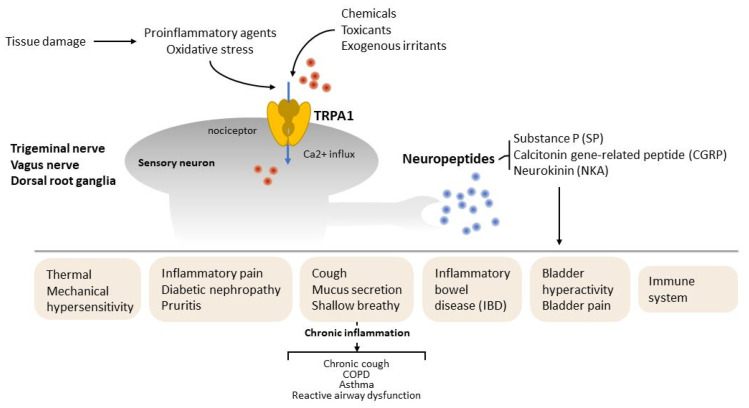
The role of TRPA1 on neurogenic inflammation. TRPA1 is expressed by sensory afferents with cell bodies in the vagal nerve, trigeminal ganglia and dorsal root ganglia that innervate peripheral targets. TRPA1 channel was activated by endogenous oxidative stress or proinflammatory agents after tissue damage. Chemicals, toxicants and exogenous irritants released neuropeptides, such as substance P (SP), calcitonin gene-related peptide (CGRP) and neurokinin A (NKA), to regulate tissue injury and inflammation. Red dots indicate Ca^2+^ and blue dots indicate neuropeptides.

Previous research focused on the role of TRPA1 in the regulation of neuropeptide release and neurogenic inflammation. In mammals, TRPA1 is widely expressed in sensory afferents that have cell bodies in nodose, dorsal root and trigeminal ganglia and innervate peripheral targets, including the skin and viscera [34,35]. TRPA1 channel activation by exogenous irritants, chemicals and proinflammatory agents is required for the release of neuropeptides, such as substance P (SP), calcitonin gene-related peptide (CGRP) and neurokinin A (NKA) [36,37] to promote and modulate inflammatory responses (Figure 2).

### 3.1. The Role of TRPA1 in Neurogenic Inflammation

In cold and inflammatory pain, a clear link exists between TRPA1 activation and inflammatory hypersensitivity. For example, AITC, an irritant in wasabi and other *Brassica* plants [35], can directly activate the TRPA1 channel and then trigger the release of SP and CGRP to promote thermal and mechanical hypersensitivity. TRPA1 is required for hypersensitivity in inflammatory pain models [12]. Pharmacological blockage or genetic knockout of TRPA1 significantly attenuates hypersensitivity [38,39]. Several studies showed the role of TRPA1 in diabetic peripheral neuropathy; neuropathic tissue produces ROS to upregulate TRPA1 in the dorsal root ganglion (DRG) and results in nociceptor sensitisation [40,41,42]. Inflammatory pain studies extend to dental [43], postsurgical [44], muscle pain [45], migraine [46] and arthritis [47]. In addition, pruritus is associated with many inflammatory conditions and the histamine-related signalling pathway [48,49]. Several studies have indicated the function of TRPA1 in the mediation of histamine-independent or nondependent pruritis [50,51,52].

In airway inflammation, the respiratory tract is innervated by TRPA1-expressing primary afferent fibres from the trigeminal nerve, vagal nerve and DRG [53,54]. Numerous exogenous irritants and endogenous mediators of airway inflammation activate the TRPA1 and further lead to the release of inflammatory neuropeptides [55], which induce bronchoconstriction, vasodilation, recruitment of the immune cells and modulation of the inflammatory response. These effects promote protective physiological responses, such as coughing, increased mucus secretion and shallow breathing [56]. However, chronic inflammation by these insults results in the development of diseases, such as chronic cough, chronic obstructive pulmonary disease, asthma and reactive airway dysfunction syndrome [57,58,59].

In gastrointestinal inflammation, the gastrointestinal tract is innervated by TRPA1-expressing primary afferent fibres from DRG that can detect inflammatory agents in the gastrointestinal tract and mediate inflammatory hypersensitivity to these stimuli via the regulation of neuropeptides release [60]. Experimental colitis models induced by 2,4,6-trinitrobenzene sulfonic acid or dextran sodium sulphate cause hypersensitivity to colorectal distension and pain [61,62]. Upregulation of TRPA1 was reported in patients with inflammatory bowel disease (IBD) [63], but the role of TRPA1 in IBD is still controversial. Engel et al. demonstrated that the activation of TRPA1-expressing vagal sensory neurons evokes a proinflammatory effect in the gut by releasing SP, and the blockade of TRPA1 decreases colitis [64]. However, Kun et al. reported the protective role of TRPA1 activation in the colonic inflammatory response [63].

In the low urinary tract, TRPA1 is expressed in neuronal fibres that innervate the bladder and urethra and is also expressed in urothelial cells. They are involved in low urinary tract nociception and mechanosensory transduction [65]. TRPA1 can regulate bladder pain and overactivity via the release of neuropeptides; an antagonist of TRPA1 can alleviate bladder hyperalgesia in cystitis and bladder pain [66,67]. Therefore, TRPA1 is implicated in the pathology of overactive bladder and associated with spontaneous and involuntary bladder contractions in spinal cord injury [68].

TRPA1 plays a specific role in the modulation of innate immunity, which can detect and respond to noxious bacterial and viral materials. TRPA1 in vagal and somatic nociceptors when rapidly activated by lipopolysaccharide (LPS) or endotoxin can cause pain, neurogenic inflammation and vasodilation due to the release of local neuropeptides [69]. The mechanism of TRPA1 activation by LPS remains unclear. A study demonstrated that a correlation exists among structural features in lipid A, the biologically active lipid moiety in LPS and TRPA1 activation in vitro. Another study speculated that LPS insertion in the bilayer of TRPA1 alters membrane tension and opens the TRPA1 channel [70,71]. Peripheral nervous and immune systems represent the main sensory interfaces between the internal milieu and the external environment, and TRPA1 may play an important role in danger detection and neuroimmune interactions.

### 3.2. The Role of TRPA1 in Non-Neurogenic Inflammation

Recent TRPA1 studies extended to the non-neuron cells [72]. The TRPA1 channel is widely expressed in many different cell types, including keratinocytes and fibroblasts [73,74], odontoblasts and dental pulp [75], chondrocytes and synoviocytes [76], enterochromaffin cells [77], vascular endothelium [78], urothelium [79], cornea [80], lung fibroblasts, smooth muscle cells and epithelial cells [81] and cardiomyocytes and cardiac fibroblasts [82,83]. A physiological or pathophysiological role for non-neuronal TRPA1 is related to inflammation, infection and immunity. Keratinocytes and different types of fibroblasts express TRPA1, which can stimulate cutaneous inflammation via cytokine or prostaglandin release and maintain the integrity of the immune response in the skin [74,84]. Odontoblasts originate from the outermost layer of the dental pulp and are responsible for dentin formation. Human odontoblasts expressing TRPA1 may act as nociceptors to detect noxious cold stimuli in teeth and mediate ATP release [75,85]. TRPA1 is upregulated after exposure to lipopolysaccharide in dental pulp cells via P38/MAPK signalling to promote differentiation and mineralisation [86]. TRPA1 is functionally expressed in synovial cells and fibroblasts [87,88] that mediate the production of arthritis-related proinflammatory factors or cytokines to lessen pain and to slow the progression of arthritis [87,89]. TRPA1 channel is widely expressed in enterochromaffin cells in the intestine and acts as a chemo-sensor to regulate gastrointestinal motility via serotonin release [90,91], thereby alleviating constipation and visceral pain. In the vasculature, cerebral endothelial TRPA1 can mediate the vasodilatory response by ROS-dependent mechanism and monitor local oxidant and redox status in the brain to regulate vascular flow and nutrient availability [92,93]. The TRPA1 channel is expressed in the urothelial and smooth muscle cells in the bladder mucosa that function as pathophysiological bladder sensory detector and regulate bladder contraction [94,95,96]. In injured cornea, the loss of TRPA1 expression or the blockade of its activity can alleviate corneal inflammation to reduce fibrosis and scarring [97]. In the respiratory tract system, TRPA1 is localised to non-neuronal airway cells, including fibroblasts and epithelial and smooth muscle cells, and it promotes non-neurogenic inflammation [98]. The stimulation of the lung epithelial cells by cigarette smoke, a major oxidant, increases the TRPA1 mediated production of IL-8. In mice, epithelial TRPA mediates lung inflammation due to cigarette smoke [99,100]. TRPA1, expressed in cardiac myocytes, plays a role in the regulation of myocardial reperfusion injury. TRPA1 activators reduce myocardial injury in the rat ischemia reperfusion (IR) model and reduce cardiac myocyte death during in vitro hypoxia-reoxygenation [101]. However, another study revealed that the genetic ablation of TRPA1 significantly decreased myocardial infarction after IR in mice. Functional TRPA1 in cardiomyocytes contributed to the release of acrolein, an IR-associated toxin, which induced Ca^2+^ overload and hypercontraction. These data indicated that the IR activation of TRPA1 worsens myocardial infarction [102]. The role and mechanism of TRPA1 in myocardial IR are conflicting and are still controversial.

In addition, TRPA1 is also expressed in macrophages [103], in which its function is regulatory along with the other nociceptors. TRPA1 can participate in ATP-induced oxidative stress and inflammation in human acute monocytic leukaemia cell line-derived macrophages [104]. TRPA1, when upregulated in atherosclerosis plaque, regulates the macrophages towards an inflammatory phenotype and alleviates atherosclerosis [105]. TRPA1 expression is increased in macrophage foam cells in the atherosclerotic aortas of apoE^−/−^ mice. The chronic administration of the TRPA1 antagonist or genetic ablation increased atherosclerosis, and the chronic administration of the TPRA1 agonist decreased the lesion. TRPA1 may be an important regulator in the pathogenesis of the atherosclerosis and cholesterol metabolism of macrophage foam cells [106]. However, TRPA1 in macrophages does not always play a protective role in cardiovascular disease. An antagonist of TRPA1 can protect cardiac hypertrophy and improve cardiac function via Ca^2+^-dependent signal pathways and inhibition of the M2 macrophages transition [107]. In other organs, the TRPA1 agonist, cannabichromene, exerts an anti-inflammatory response in activated macrophages by inhibiting nitric oxide production to ameliorate murine colitis [108]. The TRPA1 agonist cinnamaldehyde can modulate the LPS-induced systemic inflammatory response syndrome through TRPA1-dependent and TRPA1-independent mechanisms [109].

## 4. Role of TRPA1 in Kidney Disease

Little is known about the role of TRPA1 in kidney disease. Therefore, we reviewed and organised recent studies on role of TRPA1 in kidney disease, as shown in Table 1. The role of TRPA1 in kidney disease as mediator of neurogenic inflammation via sensory afferents has not been reported. The role of TRPA1 in kidney disease in published studies focused on the role of TRPA1 in non-neuronal cells. Zhu et al. demonstrated that TRPA1 prevented sepsis-induced kidney injury and improved survival in mice. A septic kidney injury model was created by caecal ligation and puncture (CLP). The study focused on the effect of TRPA1 in mitochondrial dynamics, mitochondrial biogenesis and mitophagy. TRPA1 inhibited mitochondrial mitosis, promoted fusion and enhanced the haemostasis of mitochondria. Therefore, TPRA1 downregulated CLP-induced oxidative stress in mitochondria and lessened the subsequently release of inflammatory cytokines [110]. In addition to renal TRPA1 in mitochondria, the role of TRPA1 in macrophages has been discussed in two previous studies. Ma et al. demonstrated that the knockout of TRPA1 exacerbated angiotensin II (Ang-II)-induced kidney injury in mice via the mechanism of macrophage-mediated inflammation. Ang-II can induce hypertension and kidney injury in mice, thereby mimicking hypertensive kidney disease. In the study, mRNA and protein expression of TRPA1 in kidney tissue was reduced by Ang-II. Furthermore, the knockout of TRPA1 enhanced Ang II-induced renal macrophage deposition and worsened Ang II-induced renal inflammation in mice. The activation of TRPA1 suppressed macrophage activation and induced macrophage apoptosis in vitro [111]. Ma et al. also demonstrated that the knockout of TRPA1 exacerbates renal ischemia–reperfusion injury (IRI) in mice. The protein level of renal TRPA1 was decreased by renal IRI. The knockout of TRPA1 exacerbated IRI-induced renal dysfunction and tubular injury in mice. The knockout of Trpa1 enhanced the IRI-elicited classical activation of macrophages, especially M1 macrophages, and finally enhanced IRI-induced renal inflammation [112]. Furthermore, TRPA1 is expressed in the renal tubular epithelium cells. However, the role of TRPA1 in renal cells remains unclear [113,114]. Wu et al. showed that TRPA1 was upregulated in the renal tubules of patients with acute tubular necrosis and was positively associated with oxidative stress marker. Tubular TRPA1 expression showed significant positive correlation with the severity of tubular injury. The incidence of the complete recovery of kidney function was low in patients with AKI who have high TRPA1 expression in renal tubules. Patients with high expression of TRPA1 in the renal tubule were highly likely to show nonrecovery of the renal function, which hinted that renal tubular TRPA1 was a risk factor for the recovery of renal function from acute tubular necrosis (ATN) [115]. Wu et al. demonstrated that in vivo renal IR increases tubular TRPA1 expression in wild-type mice and in vitro hypoxia–reoxygenation increases TRPA1 expression in renal tubular cells. Trpa1^−/−^ mice showed less IR-induced tubular injury, inflammation and dysfunction in kidneys compared with the WT mice. H/R evoked a ROS-dependent TRPA1 activation by Ca^2+^ influx, increased NADPH oxidase activity, activated MAPK/NF-kB signalling and finally increased IL-8 [116]. Accordingly, TRPA1 might exert different effects on renal epithelium and macrophages in renal inflammation and disease. A genetically modified mouse model with cell-specific deletion of TRPA1 is required to assess the functional role of TRPA1 in kidney disease.

## 5. Conclusions

Tissue damage can induce a series of inflammatory responses at the site of an injury. The TRPA1 channel is a molecular sentinel of tissue damage and an inflammation gatekeeper in both neuron and non-neuron cells. Kidney disease occurs due to tissue damage that is related to inflammation. Recently published research investigated the role of TRPA1 in non-neuronal cells in the kidney. In vivo TRPA1 can prevent sepsis-induced renal injury by enhancing mitochondrial haemostasis and decreasing Ang II-induced or renal ischemia-reperfusion injury through the downregulation of macrophage-related inflammation. However, renal tubular epithelial TRPA1 is an oxidative stress sensor which mediates hypoxia–reoxygenation injury in vitro and ischaemia–reperfusion-induced kidney injury in vivo through MAPKs/NF-kB signalling. It is also a risk factor for the recovery of renal function from AKI patients with ATN. The role of renal tubular TRPA1 is detrimental for these models. Hence, further investigation is warranted.

## Figures and Tables

**Table 1:** Review of previous articles on TRPA1 in kidney disease.

Authors	Year	Research	Object	Cellular Location	Injured Model	Effect	Inflammation and Oxidative Stress	Results
Zhu et al. [110]	2018	Basic	Mice	Mitochondria	Septic kidney injury	Protective	Decreased	TRPA1 prevented sepsis-induced renal injury by inhibiting mitochondrial mitosis and enhancing mitochondrial hemostasis.
Ma et al. [111]	2019	Basic	Mice	Macrophages	Ang II -induced kidney injury	Protective	Decreased	TRPA1 prevented Ang-II induced kidney injury via the downregulation of macrophage-mediated inflammation.
Ma et al. [112]	2020	Basic	Mice	Macrophages	Renal IRI	Protective	Decreased	TRPA1 prevented renal IRI via the downregulation of macrophage-mediated inflammation.
Wu et al. [115]	2019	Clinical	Human	Tubular epithelium	AKI with ATN	Potential detrimental	Correlated with TRPA1 expression	TRPA1 expression positively correlated with the severity of tubular injury. AKI patients with high expression of tubular TRPA1 had low complete renal recovery.
Wu et al. [116]	2021	Translational	In vitro, mice, and human	Tubular epithelium	Hypoxia-reoxygenation, renal IRI	Detrimental	Increased	Renal tubular epithelial TRPA1 acts as an oxidative stress sensor to mediate ischemia-reperfusion-induced kidney injury through MAPKs/NF-κB signaling

Ang-II = angiotensin II, AKI = acute kidney injury, ATN= acute tubular necrosis, IRI = ischemia-reperfusion injury.

## Data Availability

Not applicable for studies not involving humans or animals.

## Abbreviations

AKI	Acute kidney injury
CKD	Chronic kidney disease
ESRD	End-stage renal disease
TRPA1	Transient receptor potential ankyrin 1
TRP	transient receptor potential channel
ROS	Reactive oxygen species
GPCRs	G protein-coupled receptors
SP	Substance P
CGRP	Calcitonin gene-related peptide
NKA	Neurokinin A
DRG	Dorsal root ganglion
IBD	Inflammatory bowel disease
LPS	Lipopolysaccharide
IR	Ischemia-reperfusion
CLP	Caecal ligation and puncture
Ang-II	Angiotensin II
IRI	Ischemia–reperfusion injury
ATN	Acute tubular necrosis
